# In Situ Detection of Interfacial Flow Instabilities in Polymer Co-Extrusion Using Optical Coherence Tomography and Ultrasonic Techniques

**DOI:** 10.3390/polym13172880

**Published:** 2021-08-27

**Authors:** Alexander Hammer, Wolfgang Roland, Maximilian Zacher, Bernhard Praher, Günther Hannesschläger, Bernhard Löw-Baselli, Georg Steinbichler

**Affiliations:** 1 Institute of Polymer Extrusion and Compounding, Johannes Kepler University Linz, Altenberger Strasse 69, 4040 Linz, Austria; wolfgang.roland@jku.at (W.R.); maximilian.zacher@pro2future.at (M.Z.); bernhard.loew-baselli@jku.at (B.L.-B.); georg.steinbichler@jku.at (G.S.); 2 Pro2Future GmbH, Altenberger Strasse 69, 4040 Linz, Austria; 3 MoldSonics GmbH, Hafenstrasse 47-51, 4020 Linz, Austria; bernhard.praher@moldsonics.at; 4 RECENDT GmbH, Altenberger Strasse 69, 4040 Linz, Austria; guenther.hannesschlaeger@recendt.at; 5 Institute of Polymer Injection Molding and Process Automation, Johannes Kepler University Linz, Altenberger Strasse 69, 4040 Linz, Austria

**Keywords:** co-extrusion, interfacial flow instabilities, detection criterion, quantification, comparison of detection methods

## Abstract

Co-extrusion is a widely used processing technique for combining various polymers with different properties into a tailored multilayer product. Individual melt streams are combined in a die to form the desired shape. Under certain conditions, interfacial flow instabilities are observed; however, fundamental knowledge about their onset and about critical conditions in science and industry is scarce. Since reliable identification of interfacial co-extrusion flow instabilities is essential for successful operation, this work presents in situ measurement approaches using a novel co-extrusion demonstrator die, which is fed by two separate melt streams that form a well-controlled two-layer co-extrusion polymer melt flow. An interchangeable cover allows installation of an optical coherence tomography (OCT) sensor and of an ultrasonic (US) measurement system, where the former requires an optical window and the latter good direct coupling with the cover for assessment of the flow situation. The feasibility of both approaches was proven for a material combination that is typically found in multilayer packaging applications. Based on the measurement signals, various parameters are proposed for distinguishing reliably between stable and unstable flow conditions in both measurement systems. The approaches presented are well suited to monitoring for and systematically investigating co-extrusion flow instabilities and, thus, contribute to improving the fundamental knowledge about instability onset and critical conditions.

## 1. Introduction

Co-extrusion process technologies have been developed with the particular objective of combining various polymeric materials within a multilayer structure to obtain products with property profiles that are specifically tailored to their final application. Each individual layer makes a particular functional contribution to the final product properties, such as barrier, adhesion, mechanical strength, and chemical resistance. Co-extrusion is the state-of-the-art process for the continuous production of multilayer films, sheets, pipes, bottles, tubes, profiles, fibers, and other products, due to its technical and economic advantages over multistep lamination or coating processes. Two different co-extrusion die systems are available for combining the individual melt streams in the molten state [1]:

- In multi-manifold dies, the individual melt streams are fed separately into the extrusion die and are distributed to achieve the desired form. These melt streams are combined just before the die exit, which enables the processing of polymer melts with larger differences in melt temperature and viscosity, but at the cost of lower process flexibility and higher manufacturing costs;
- In the more commonly used feedblock dies, the individual melt streams are combined in an adapter. The multilayer melt stream is then passed to the die for distribution to produce the final shape. With feedblock dies, any number of layers can be processed, but all materials used must have similar melt temperatures and viscosities.

Fundamental knowledge of the co-extrusion processing behavior is essential to successful operation. Under certain conditions, interface distortions and irregularities can occur. Three important types of flow instability can be observed in multilayer polymer melt flows [2,3]:

- Viscous encapsulation can result from a viscosity mismatch between the polymers involved; the less viscous melt tends to encapsulate the more viscous melt due to an energetically favored state of minimal pressure loss.
- Elastic layer rearrangement is caused by viscoelastic flow properties of the melts involved. This type of layer distortion is observed even for materials with well-balanced viscosities and for identical melts that are colored differently. The elastic properties of the melt induce secondary flows perpendicular to the main flow direction that give rise to elastic layer rearrangement phenomena. With an increasing ratio of elastic to viscous properties, these phenomena become more pronounced.
- Interfacial flow instabilities are irregularities at the layer interface that result in transient local layer thickness fluctuations [4]. They are commonly categorized into (1) low-frequency wave-type instabilities, (2) high-frequency zig-zag-type instabilities and, occasionally, (3) a transition regime from stable to unstable process behavior. The onset and appearance of these types of instability are strongly dependent on the processing parameters and rheological behavior of the material combination. These interfacial irregularities can be related to products with inferior optical, physical, and mechanical quality, and defects become even more pronounced when a subsequent processing step involves stretching (e.g., blow molding, thermoforming).

This work focuses on the detection of undesired interfacial flow instabilities that lead, for example, to optical defects and mechanical weak spots in a multilayer polymer product. Insights into the influence of flow properties, polymer melt rheology, and processing parameters on the occurrence of interfacial flow instabilities are of high scientific and industrial interest. Although numerous studies have investigated this type of co-extrusion flow instability experimentally and theoretically, neither generally valid, consolidated, and proven criteria, nor critical values, have been published. Schrenk et al. [5] proposed the existence of a critical interfacial shear stress level that gives rise to interlayer distortions. This conclusion is based on a combination of (1) visual observation of cross-sectioned samples from die-freezing experiments of symmetrical three-layer co-extrusion die flows by six “human judges”, and (2) numerical flow analysis. Similar analyses were conducted by Han and Shetty [6,7], who investigated symmetrical three-layer and five-layer flows undergoing quenching in water after extrusion. Theoretical flow analysis indicated that further process parameters (e.g., viscosity ratio and elasticity ratio) impact the occurrence of interfacial instabilities. Mavridis and Shroff [8] used narrow-angle scattering (NAS) and viscous flow analysis (of viscoelastic material properties) to quantify interlayer instabilities in co-extrudates, and proposed reducing them by minimizing interfacial shear stress and matching elasticities of adjacent melt layers. More recently, Bondon et al. [9] and Vuong et al. [10] classified transparent co-extruded barrier films for packaging applications to investigate the effects of interfacial reactions of tie layers on interfacial flow instabilities. For that purpose, optical microscopy and an optical test to quantify transparency involving “human operators” were used.

Based on the visual appearance of extruded bilayer blown films, Tzoganakis and Perdikoulias [11] found that the molecular weight distributions of the polymers, interfacial shear stresses, extensional deformation at the merging point of the minor layer, and viscoelastic properties of the melts affect the development and extent of interfacial distortions. Zatloukal et al. [12,13,14,15] built on this work, and conducted viscoelastic multiphase simulations of co-extrusion processes and corresponding co-extrusion experiments. They proposed that wave-type interfacial instabilities can be related to normal stress differences across the interface in the confluent die area, and suggested that the onset of instability can be predicted by the “total normal stress difference (TNSD) sign criterion”.

Wilson and Khomami [16] developed a co-extrusion die equipped with several laterally fused silica glass windows for the unbiased online investigation of flow instabilities in co-extrusion processes. By means of flow visualization using digital image processing, the effects of viscoelasticity and of introducing regular periodic disturbances on the emergence, growth, and decay of interlayer distortions were monitored and compared to theoretical stability analysis results [16,17,18]. Similarly, Martyn et al. [19,20] employed lateral borosilicate glass windows in an experimental die and a charge-coupled device (CCD) camera to observe interlayer instabilities during the co-extrusion of identical and dissimilar melts. Furthermore, the stress fields were evaluated by analyzing flow-induced stress birefringence patterns, which showed excellent agreement with viscoelastic finite element simulations. Their results indicate that distortions are more pronounced at higher melt elasticities, and cannot be attributed to differences in surface energies and small perturbations in the extrusion process.

Zhang et al. [21] reported that interfacial defects may be due to the continuous development of a diffuse interphase triggered by the interpenetration of macromolecules during the extrusion process. Stable co-extrusion of highly compatible polymers (resulting in thicker interdiffusion layers) is therefore feasible even at high interfacial elasticity and viscosity ratios. However, they did not present any underlying causes of the effects observed.

Most of the published experimental work in this context is based on inspecting the extruded melt film or solidified extrudate, where the former approach is limited to transparent melts and is highly subjective, while the latter is time-consuming and no information about the flow situation is available during co-extrusion. Hence, in this work, we present various in situ measurement methods for detecting interfacial flow instabilities in polymer co-extrusion. We investigated:

- Optical coherence tomography (OCT);
- Ultrasound measurements.

First, we analyzed the problem and evaluated the theoretical suitability of these two approaches for detecting the interface in multilayered polymer structures. On this basis, we evaluated their ability to detect small fluctuations in interface position caused by flow instabilities. Both approaches were then tested experimentally in a novel two-layer co-extrusion demonstration die that we designed specifically to investigate flow instabilities under controlled conditions. The die is equipped with a glass insert to provide an optical window for OCT measurements. For both approaches, we present testing and evaluation procedures that allow for the characterization of interfacial flow instabilities.

## 2. Technologies for In Situ Detection of Interfacial Flow Instabilities

### 2.1. Key Requirements for Co-Extrusion Die and Detection Technology

As presented above, several approaches to investigating interfacial co-extrusion instabilities can be found in the literature. These can be subsumed under the following categories, which are characterized by several advantages and disadvantages with respect to the technical design of the die and the process and material combinations being investigated:

- Analysis of frozen cross-sections requires adequate sample preparation, and is a time-consuming offline method—which means that information on flow stability is not instantly available—and is therefore unsuitable for process control;
- Visual analysis of the extruded melt stream is the simplest detection method, but requires at least one melt of the co-extrudate to be transparent. It is also biased, since the evaluation depends heavily on the observer and the environment (e.g., ambient light, lighting, and point of observation);
- Visual analysis of the melt flow in the die through optical viewports using various detection systems involves modifications to die geometry and materials (e.g., insertion of lateral silica glass windows), whose effects on co-extrusion flow properties (e.g., wall slippage because the coefficients of friction differ from that of steel) may have to be taken into account. Additionally, a lateral view may lead to misinterpretation, because potential secondary flow effects are most pronounced at the channel edges of flat film flow geometries.

Hence, a holistic perspective on co-extrusion die design, the polymeric materials involved, and specifications of the sensor equipment is needed. In developing such an in situ system for detecting interfacial co-extrusion instabilities, we considered the following requirements:

- The measurement system must be applicable to a wide range of material combinations, including amorphous and semicrystalline polymers;
- Material modifications (e.g., by compounding with optically active particles) are to be avoided;
- Detection must be in situ in order to enable real-time evaluation of flow instabilities;
- The detection system must resist thermal load resulting from heat radiation and heat conduction from the die;
- The signal intensity must be sufficient to penetrate through the die wall and the melt stream;
- Adequate depth resolution of the sensor is required to detect low-amplitude instabilities;
- An objective and reliable classification criterion must be developed that quantifies and assesses the stability of the co-extrusion flow;
- The measurement technique must be robust and yield reproducible results.

### 2.2. Optical Coherence Tomography (OCT)

For over 25 years, OCT has been enriching the world of optical imaging methods [22,23]. Considerable progress has been made both technologically and in applications. OCT has been used not only in important medical applications in ophthalmology [24] and dermatology [25], but also in an increasing number of industrial measurement tasks [26,27,28].

**Figure 1:**
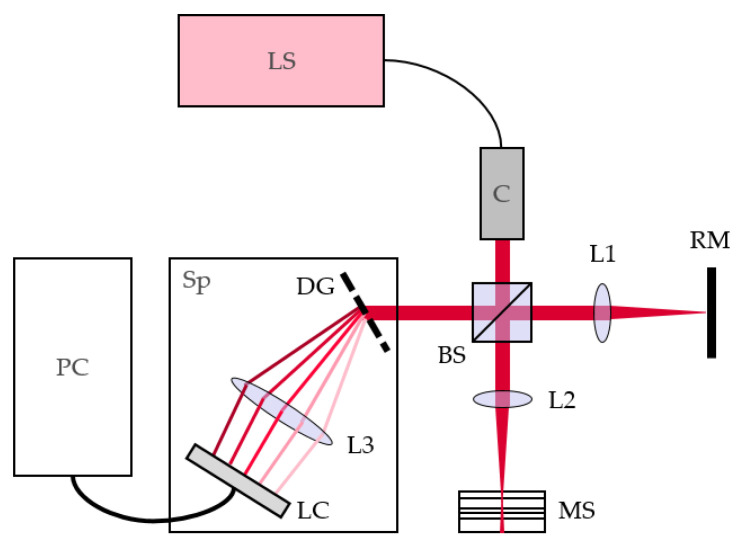
OCT principle and components: light source (LS); collimator (C); beam splitter (BS); focusing lenses (L1, L2); spectrometer lens system (L3); reference mirror (RM); multilayer sample (MS); spectrometer (Sp); diffraction grating (DG); line camera (LC); computer (PC).

OCT is an optical, non-contact, non-destructive, high-speed imaging method; it uses low-coherence interferometry for the high-resolution measurement of transparent or semi-transparent samples. Due to substantial improvements in ruggedness and more compact design, OCT is very well suited to in-line monitoring in an industrial environment [29]. In flow instability detection, OCT offers advantages in terms of speed and sensitivity. Figure 1 shows the key components of OCT and how they are connected. Incoming light passes through a Michelson-type interferometer. Light reflected by the reference mirror interferes with light reflected by the sample (both at the surface and inside), and is analyzed using the spectrometer. The resulting spectra are computer-processed to obtain depth-scan information.

### 2.3. Ultrasound

Ultrasound-based techniques have long been used in various medical applications (e.g., cardiology and ophthalmology) for diagnostic imaging and therapy. Ultrasonic systems are also employed in a wide range of industrial contexts, including flow-rate measurement, distance measurement, and material treatment. In the field of polymer processing, in situ characterization of polymer melts during processing is mainly used in research, but is attracting increasing industrial interest. Areas of application include measurement of melt temperature [30], melt inhomogeneity [31], melting behavior along the plasticizing screw [32,33], and density measurements [34]. Furthermore, there are applications in the field of condition monitoring, such as screw wear measurement [35]. Measurement of flow instabilities during co-extrusion is an interesting new field of application for ultrasonic systems in polymer processing.

A typical ultrasonic system, as used in polymer processing, consists of a pulser-receiver unit and the associated ultrasonic sensors. The pulser-receiver unit generates a short (~µs) high-voltage pulse that is sent to the ultrasonic sensor, where a piezoelectric element converts the voltage pulse into an ultrasonic pulse that passes through the object under examination. Either the reflections from the boundary layers are reflected and captured by the same sensor (reflection measurement), or the pulse passes through the structure and is received at an opposing sensor (transmission measurement). The ultrasound signal is converted into an electrical signal by the sensor’s piezoelectric element. In the pulser-receiver unit this signal is filtered, amplified, and finally digitized. The pattern of signals caused by the interaction between ultrasound and the polymer melt and its boundary layers allows a variety of properties of the polymer melt to be investigated (e.g., structure, temperature, density, and thickness).

## 3. Experimental

### 3.1. Materials

Plastic products with properties that provide a barrier against moisture and oxygen play a significant role in the packaging industry. Such films, sheets, and extrusion blow-molded hollow articles are commonly processed using co-extrusion techniques to combine various polymers within a multilayer structure that comprises one or more base polymer layers (e.g., polyolefins), a barrier layer, and adhesive layers. We chose to investigate a polymer combination of a high-density polyethylene (HDPE) and a maleic-anhydride-grafted, polyethylene-based adhesive resin in this work. This combination is often used in industrial co-extrusion processes, and potentially results in unstable interfaces under certain processing conditions. The supplier, main application, density, and melt flow rate (MFR) of the two materials obtained from [36,37] are listed in Table 1.

**Figure 2:**
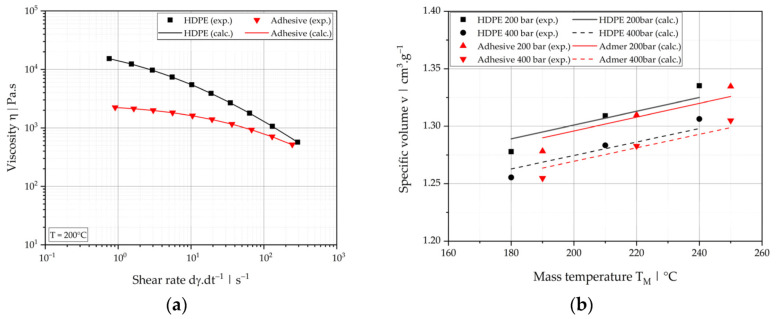
Process-relevant material properties: (**a**) viscosity data at *T* = 200 °C and (**b**) pvT data at *p* = 200 bar and 400 bar for HDPE ACP5831D and adhesive NF408E.

To determine the shear rate and temperature-dependent viscosity, a HAAKE Rheomex OS system (Thermo Fisher Scientific, Waltham, USA) was used that consisted out of a single-screw extruder with a screw diameter of 19 mm and a screw length of 33 times the diameter, a melt pump (2.4 cm^3^/rev.), a bypass valve, and a slit die (gap height of 1.2 mm and a width of 20 mm) equipped with three pressure transducers and a melt temperature sensor. Rheological measurements were conducted at apparent shear rates within a range between 0.8 and 290 s^−1^ and die temperatures of 180 °C, 200 °C, and 220 °C. Apparent shear rates and wall shear stresses were corrected with the Weissenberg-Rabinowitsch correction, as applied in [38]. The experimentally derived viscosity data were approximated using a Carreau-Yasuda model [39,40]:

(1) $$\eta_{CY}\left(\dot{\gamma },T\right)=a_{T}\eta_{\infty }+a_{T}\left(\eta_{0}-\eta_{\infty }\right){\left(1+{\left(a_{T}\lambda \dot{\gamma }\right)}^{a}\right)}^{\frac{n_{CY}-1}{a}},$$

with $\eta_{0}$ and $\eta_{\infty }$ denoting the viscosities at zero shear rate and infinite shear rate, respectively. Furthermore, $n_{CY}$ is the Carreau-Yasuda power-law index, and λ is the relaxation time. The temperature dependence of viscosities was considered using an approximate Arrhenius approach by evaluating the temperature shift factor $a_{T}$ for each measurement point and its measured melt temperature:

(2) $$\;a_{T}=\exp\left(-\alpha \left(T-T_{0}\right)\right),$$

where α is the material-specific temperature sensitivity of the viscosity and $T_{0}$ is the reference temperature. The Carreau-Yasuda model parameters for the two materials are summarized in Table 2, and the viscosity curves at 200 °C and corresponding experimental data points are presented in Figure 2a.

The specific volume as a function of pressure and temperature was measured using a RHEOGRAPH 25 high-pressure capillary rheometer (Göttfert, Buchen, Germany). The pressure-volume-temperature behavior (pvT) was then approximated using a Tait equation [41,42], considering only specific volumes in the upper temperature region (above the transition temperature, Equation (3)). The Tait parameters for the materials are listed in Table 3, and example predictions and experimental data points are shown in Figure 2b.

(3) $$v\left(p,T\right)=\left[b_{1m}+b_{2m}\left(T-b_{5m}\right)\right]\left[1-0.0894\ln\left(1+\left(\frac{p}{b_{3m}\;e^{-b_{4m}\left(T-b_{5m}\right)}}\right)\right)\right].$$

### 3.2. Equipment and Procedure

**Figure 3:**
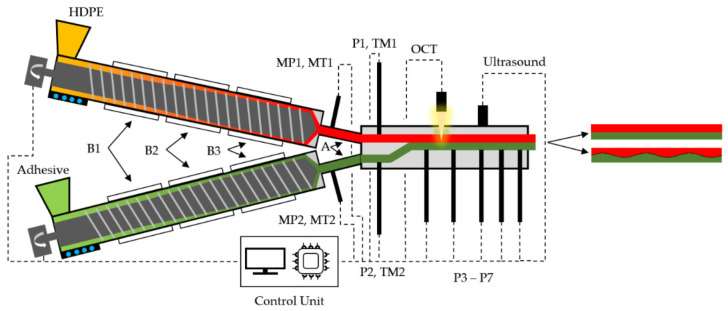
Schematic representation of the experimental setup with the upper-layer extruder for plasticating the HDPE, the bottom-layer extruder for plasticating the adhesive, the co-extrusion demonstration die with in situ measurement equipment, the control unit, and additional sensor equipment: barrel heating zone (B1 to B3), adapter (A), back pressure transducer (MP1, MP2), melt temperature sensors (MT, TM), and melt pressure transducers (P1 to P7).

In this work, we used a two-layer lab-scale co-extrusion line, as shown schematically in Figure 3. We designed the key component—a novel two-layer co-extrusion demonstration die—with the particular objective of investigating interfacial flow instabilities. The two melt streams are combined within a feedblock section, which is followed by a stratified flow region with a constant rectangular cross-section. This geometry enables controlled co-extrusion flow conditions that can be described accurately without the need for complex three-dimensional computational fluid dynamics (CFD) simulations. To enhance thermal and material homogeneity, static mixing elements are placed within the adapter system in front of the feedblock of the die. Furthermore, a melt temperature sensor at each die inlet (TM1 and TM2) and seven pressure transducers (P1 to P7) along the axial flow path are positioned to characterize melt temperatures and pressure behavior within the die. Temperature control of the die is achieved by heating cartridges and temperature sensors in each heating zone, which were all set to a constant temperature as listed in Table 4. To be able to perform both OCT and ultrasonic measurements, two exchangeable versions of the co-extrusion die cover were developed: a massive steel cover for ultrasonic measurement, and a cover with a quartz glass insert for OCT measurement.

The materials were plasticated using two identical ECE-Co-Extruder-30–18 kg/h single-screw extruders (extrunet, Eberstalzell, Austria) with smooth barrels, with a diameter of *D* = 30 mm and an axial length of *L* = 606 mm (*L* = 20.2 *D*). Furthermore, the extruders were equipped with square-pitched, single-flighted three-zone screws with a compression ratio of 2.32. Pressure transducers (MP1 and MP2) and mass temperature sensors (MT1 and MT2) were positioned immediately after the screw tip of each extruder to measure back pressure and melt temperature, respectively. Thermal energy was supplied by electrical heaters in the three barrel zones (B1 to B3) and the adapter zone (A), which connected the extruders to the co-extrusion die. The system was cooled with water in the feed housing and forced air in the barrel zones. The barrel and adapter temperature profiles for the materials investigated are listed in Table 4.

For a particular polymer melt combination, two-layer co-extrusion flow processes in rectangular ducts can be directly influenced by means of throughput and melt temperature. In developing and testing the sensor technologies for detecting interfacial flow instabilities, we did not consider the influence of individual melt temperatures on the flow process (and, thus, on materials’ viscosities). Consequently, we investigated certain operating points while varying the overall throughput $\dot{m}$ and the ratio of individual throughputs. The position of the interface can thus be changed, which results in either stable or unstable processes. Table 5 gives an overview of the screw rotational speeds of the operating points investigated. We aimed at developing a functioning detection method for interfacial instabilities on a maximum range of severity. By using these screw rotational speeds, operating points ranging from a distribution of approximately 50:50% of HDPE and adhesive up to a highly asymmetric flow can be investigated (expected to result in stable and highly unstable interfaces, respectively). A higher density of operating points was defined around the region of transition from stable to unstable flow behavior to evaluate the sensitivity of the methods (see Section 4).

Experiments were carried out by (1) setting the screw speeds, (2) waiting for equilibrium process conditions, (3) collecting pressure and temperature data for at least 10 min at a sampling rate of 50 ms, (4) monitoring the stability of the flow with the in situ sensor equipment for intervals of 10 s (OCT) and 7.125 s (US), and (5) evaluating process and OCT/ultrasonic data collected. Since OCT and ultrasound measurements require different setups of the co-extrusion die, this experimental procedure was repeated for both technologies and all operating points. As transforming optical distances between interfaces obtained from OCT measurements into real distances requires the refractive index of the material involved, we conducted two additional OCT experiments by co-extruding two identical layers of (1) HDPE and (2) adhesive through the co-extrusion die in order to determine the polymers’ refractive indices in the molten state.

### 3.3. Flow Modeling

To predict the exact position of the interface within the co-extrusion flow of adhesive and HDPE through the die with rectangular cross-section, we applied a symbolic regression model developed as part of our previous work [43,44]. Using a power-law model according to Ostwald and de Waele to consider the shear rate dependency of viscosities, we found that such flow situations (and, thus, the interface position) can be fully described by a set of four independent dimensionless parameters—namely, (1) the power-law index of fluid A $n_{A}$, (2) the power-law index of fluid B $n_{B}$, (3) the ratio of dimensionless pressure gradients χ, and (4) the dimensionless volume flow rate of fluid A $\Pi_{V}^{A}$. Since this is a dimensionless modeling approach, the height of bottom-layer fluid A $h_{A}$ (adhesive) is normalized by the overall channel height h to obtain the dimensionless position of the interface κ:

(4) $$\kappa =\frac{h_{A}}{h}.$$

(5) $$\kappa =f\left(n_{A},n_{B},\chi ,\Pi_{V}^{A}\right).$$

**Figure 4:**
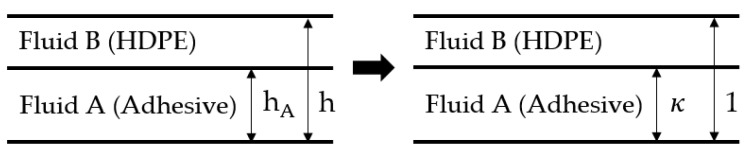
Schematic representation of the transformation of the flow channel from dimensional into dimensionless representation.

A schematic of the flow domain and its transformation into a dimensionless system is presented in Figure 4. The parameters χ and $\Pi_{V}^{A}$ can be evaluated by taking into account the flow consistencies of both polymer melts $K_{A}$ and $K_{B}$, the mean flow velocity within the channel $v_{ref}$, the volumetric flow rate of fluid A ${\dot{V}}_{A}$, and the total volumetric flow rate $\dot{V}$:

(6) $$\chi =\frac{K_{B}}{K_{A}}{\left(\frac{h}{v_{ref}}\right)}^{n_{A}-n_{B}},$$

(7) $$\mathrm{with}\;v_{ref}=\frac{{\dot{V}}_{A}+{\dot{V}}_{B}}{wh},$$

(8) $$\Pi_{V}^{A}=\frac{{\dot{V}}_{A}}{\dot{V}}.$$

As the modeling framework applied is grounded on a power-law viscosity model, the Carreau-Yasuda parameters must be transformed into their corresponding local power-law parameters. To this end, we calculate a representative shear rate within the flow channel by using the average flow velocity and overall channel height:

(9) $${\dot{\gamma }}_{rep}=\frac{v_{ref}}{h}.$$

Interpreting the power-law model as a local tangent of the Carreau-Yasuda model allows its two parameters to be determined as follows:

(10) $$n=\frac{\left(\eta_{0}-\eta_{\infty }\right)\left(n_{CY}-1\right){\left(a_{T}\lambda {\dot{\gamma }}_{rep}\right)}^{a}{\left(1+{\left(a_{T}\lambda {\dot{\gamma }}_{rep}\right)}^{a}\right)}^{\frac{n_{CY}-1-a}{a}}}{\eta_{\infty }+\left(\eta_{0}-\eta_{\infty }\right){\left(1+{\left(a_{T}\lambda {\dot{\gamma }}_{rep}\right)}^{a}\right)}^{\frac{n_{CY}-1-a}{a}}}.$$

(11) $$K=\eta_{\infty }+\left(\eta_{0}-\eta_{\infty }\right){\left(1+{\left(a_{T}\lambda {\dot{\gamma }}_{rep}\right)}^{a}\right)}^{\frac{n_{CY}-1}{a}}{\dot{\gamma }}_{rep}{}^{1-n}.$$

The main advantage of this approach is its ability to capture the upper Newtonian plateau by a local power-law approximation, thus combining both accurate predictions of viscosities and mathematically simple flow modeling. In the co-extrusion die developed in this work, average shear rates are observed in the upper Newtonian plateau or in the region of transition to shear-thinning behavior, which is also commonly the case in industrial die flows.

### 3.4. Approach to Optical Coherence Tomography Measurement

For this study, the Telesto (Thorlabs, Lübeck, Germany) spectrometer-based OCT-system was chosen, the specifications of which are given in Table 6.

The fundamental measurement performed in an SD-OCT system is a so-called A-scan (or depth scan), which captures the depth profile of the sample at the current beam position. The A-scan shows a peak at every depth position where light is reflected, which is particularly the case at the interface between two materials with different refractive indices. As the light beam is scanned along one axis across the sample, multiple A-scans form a B-scan. We found B-scans to be the ideal tool for detecting the interface between the top and bottom layers of the extruded material.

**Figure 5:**
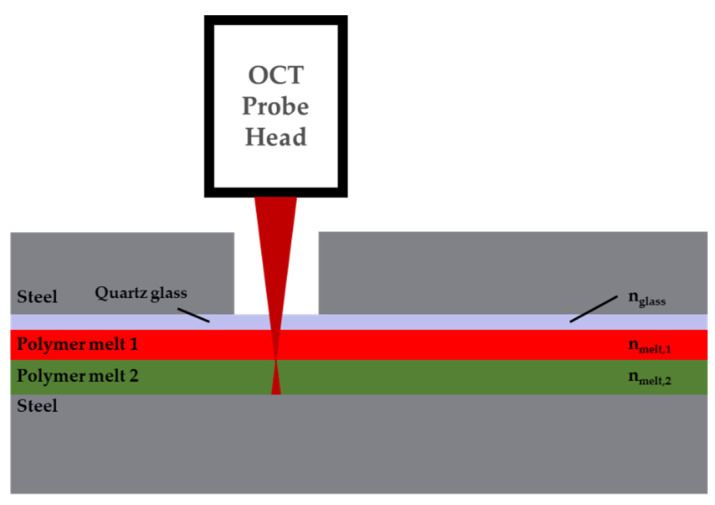
OCT measurement arrangement of OCT for the detection of flow instabilities.

Since access to the melt requires an optical viewport, we designed a new extrusion die cover with an integrated quartz glass plate. Figure 5 shows how the OCT probe head is attached to the die. Along the OCT beam there are four material interfaces that can contribute to the OCT signal: air/glass, glass/polymer 1, polymer 1/polymer 2, and polymer 2/steel. All of these signals are due to changes in the refractive index at the positions of the interfaces, where part of the beam is reflected. The main focus of these measurements is on the interface or transition between polymers 1 and 2. Another component of the OCT signal is a speckle pattern that appears inside a homogeneous layer, and is caused by multiple reflecting and scattering particles in the material. In many cases, even if the difference in refractive index is not high enough to define the interface distinctly, these speckle patterns can be used to identify the boundary between the two materials.

### 3.5. Ultrasound Measurement Approach

**Figure 6:**
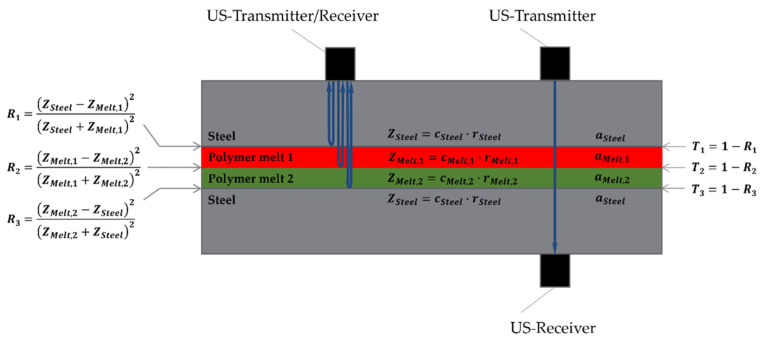
Measurement arrangement for detecting flow instabilities via ultrasound reflection and transmission.

For the ultrasound experiments, a Plast pulser-receiver unit with a sampling frequency *f* = 100 MHz and a resolution of the analog-to-digital converter (ADC) of 10 bit, and Plast Surface ultrasonic sensors with *f* = 4 MHz (Moldsonics, Linz, Austria), were employed. We identified three parameters that can be used to detect flow instabilities via ultrasound—namely, (1) the change in transit time, (2) the intensity of the ultrasonic pulses transmitted through the two co-extruded polymers, and (3) the change in the position of the interface of the two polymer melts by detection of the reflected ultrasonic pulse by this layer. The measurement setup for detecting the co-extrusion flow instabilities in reflection and transmission modes is shown schematically in Figure 6.

In reflection measurement the ultrasonic sensor is used simultaneously as transmitter and receiver, while in transmission measurement the transmitter and receiver are located opposite to one another. In both arrangements, ultrasound is coupled from the transmitter into the steel of the die and travels towards the polymer melt. The first reflection of the ultrasonic pulse occurs at the interface between steel and polymer melt 1. The proportion of reflection of the acoustic energy at the interface can be expressed by the reflection coefficient *R*_1_:

(12) $$R_{1}=\frac{{\left(Z_{Steel}-Z_{Melt,1}\right)}^{2}}{{\left(Z_{Steel}+Z_{Melt,1}\right)}^{2}}.$$

The acoustic impedances $Z_{Steel}$ and $Z_{Melt,1}$ of steel and polymer melt 1, respectively, are products of the longitudinal sound velocities $c_{L}$ and the densities ρ in the respective materials. The fraction of sound energy transmitted at the interface is thus given by *T*_1_ = 1 − *R*_1_, with $T_{1}$ denoting the transmission coefficient. Due to the substantial differences in density and sound velocity between steel and polymer melt, the proportion of sound energy transmitted into the melt is extremely low (1–3%). The next reflection occurs at the interface layer between the two polymer melts, with $R_{2}$ the reflection coefficient and $Z_{Melt,2}$ the acoustic impedance of polymer melt 2:

(13) $$R_{2}=\frac{{\left(Z_{Melt,1}-Z_{Melt,2}\right)}^{2}}{{\left(Z_{Melt,1}+Z_{Melt,2}\right)}^{2}}.$$

The reflected pulse can be detected by the ultrasonic sensor in the reflection measurement configuration. Accordingly, the change in transit time of this pulse reflection is a good indicator of the change in position of the polymer-polymer interface and, thus, of flow instabilities. The smallest changes in transit time of an ultrasound pulse in the ns range can be detected by modern high-accuracy ultrasound measurement systems. This type of measurement is challenging due to the small differences between the acoustic impedances of the two polymer melts and the very low resulting amplitude of the reflected pulse (additionally, the pulse amplitude is reduced considerably by the transition from polymer melt 1 into steel along the acoustic path back to the ultrasound transducer). Finally, the last reflection occurs at the interface between polymer melt 2 and steel, with the following reflection coefficient:

(14) $$R_{3}=\frac{{\left(Z_{Melt,2}-Z_{Steel}\right)}^{2}}{{\left(Z_{Melt,2}+Z_{Steel}\right)}^{2}}.$$

Due to the multiple resulting reflections, evaluation of the changes in intensity and time of flight of the reflected ultrasound pulses through both polymer melts—which we also identified for the detection of co-extrusion flow instabilities—require considerable effort in the data evaluation step; therefore, transmission measurement is preferable for these two parameters. As described above, all reflections occur at the interfaces; however, the pulse transmitted through the whole structure (steel, polymer melt 1, polymer melt 2, steel) is measured by the ultrasound receiver. The intensity I of the ultrasonic pulse in a medium at position x is given by:

(15) $$I\left(x\right)=I_{0}e^{-\alpha x},$$

with the initial intensity *I*_0_ = *I* (*x* = 0) and the material-specific attenuation coefficient α. Since, in our experimental setup, we consider steel temperature and polymer melt pressure and temperature to be almost constant, we can assume that changes in the received pulse amplitude (which is proportional to the intensity) are caused by changes in the thicknesses of the two polymer melts. A requirement for successful detection is that the damping coefficients of the two polymer melts be sufficiently different. When measuring the change in transit time through the steel and the two polymer melts, it is also assumed that the speed of sound in steel and in the two polymer melts is nearly constant, and that the change in transit time is caused by the change in thickness between the two polymer melts. The longitudinal sound velocity $c_{L}$ in polymer melts is given by:

(16) $$c_{L}={\left[\frac{1}{\rho \kappa }\right]}^{1/2},$$

with ρ being the density and κ the adiabatic compressibility of the polymer melt, which both depend on the temperature and pressure of the melt. It is known that the speed of sound in polymer melts depends almost linearly on pressure and temperature; it decreases linearly with increasing temperature and increases linearly with rising pressure [30]. Again, for successful measurement of flow instability, the sound velocities of the two polymer melts must be sufficiently different.

**Figure 7:**
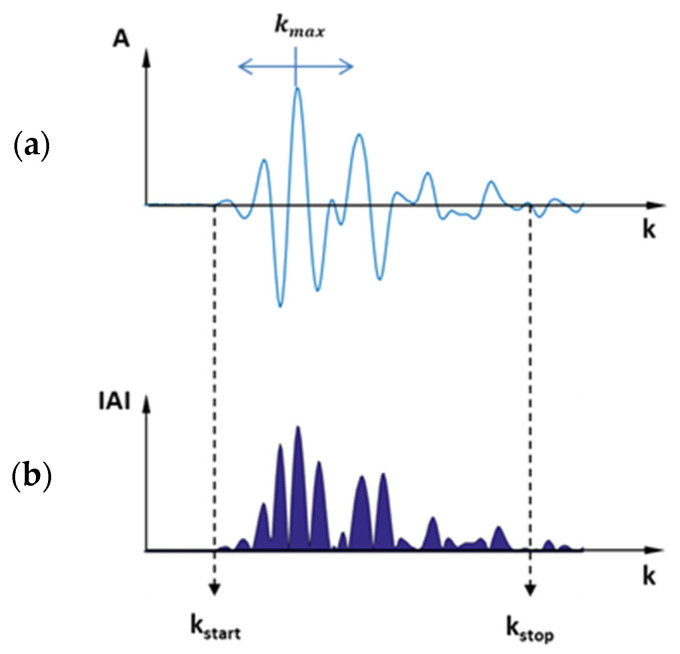
Quantities evaluated using the measured ultrasonic pulse: (**a**) sampling point $k_{max}$ and (**b**) amplitude sum $A_{sum}$.

Preliminary investigations using the reflection measurement arrangement showed that it is impossible to detect flow instabilities via the change in position of the interface layer between the two polymer melts based on the ultrasonic pulse reflected at this layer, because the acoustic impedances of the two polymer melts differ only slightly. The very low reflection coefficient resulted in an amplitude of the reflected pulse at the interface layer that was too low and could not be directly resolved with our measuring system. For this reason, transmission measurements were subsequently carried out that measured the change in transit time and/or intensity of the ultrasonic pulses transmitted through the two co-extruded polymers. Again, the relatively small changes in the thermodynamic and rheological properties of the two polymer melts resulted in very small changes in transit time and intensity when the thickness ratios of the melts were changed. Since the changes were at the limit of the detection capability of the pulser-receiver used, the linear correlation coefficient of the change in transit time with the change in intensity was found to be a parameter to successfully approximate interfacial instabilities. Figure 7 shows the two characteristic quantities of the ultrasonic pulse transmitted through the two polymer melts, which are evaluated by:

(17) $$k_{max,n}=\underset{k\in \left[k_{start},k_{stop}\right]}{\arg\;\max\;}A_{n}\left(k\right)-\underset{n\in \left[1,n_{stop}\right]}{\mathrm{mean}}\left(\underset{k\in \left[k_{start},k_{stop}\right]}{\arg\;\max\;}A_{n}\left(k\right)\right);$$

(18) $$A_{sum,n}=\overset{k_{stop}}{\underset{k=k_{start}}{\sum }}\left|A_{n}\left(k\right)\right|-\underset{n\in \left[1,n_{stop}\right]}{\mathrm{mean}}\left(\overset{k_{stop}}{\underset{k=k_{start}}{\sum }}\left|A_{n}\left(k\right)\right|\right),$$

where $A_{n}\left(k\right)$ describes the amplitude value A as a function of the sample point k of the *n*th measurement. The pulse must be within the interval between the sampling points $k_{start}$ and $k_{stop}$. The relative time of flight of the pulse of the *n*th measurement is measured by detecting the maximum of the amplitude value A at sampling point $k_{max}$. A value proportional to the pulse intensity is formulated by taking the sum of the absolute values of $A_{n}\left(k\right)$. For both values, the mean value of the respective variable over all measurements is subtracted for offset correction. The linear correlation coefficient is then calculated from these two functions by using covariance and variance:

(19) $$R=\frac{cov\left(k_{max},A_{sum}\right)}{\sqrt{var\left(k_{max}\right)}\;\sqrt{var\left(A_{sum}\right)}}.$$

## 4. Results and Discussion

The operating points investigated were characterized based on (1) the position of the polymer-polymer interface, (2) visual observation of the co-extrudate (since the two melts are highly transparent), and (3) evaluation of B-scans from OCT measurements, and correlation between the transit time and intensity of transmission mode ultrasound measurements. As we used several methods to assess the stability of the interface, we were able to cross-validate the results of our approaches to in situ detection.

In the first step, we analyzed the mass throughputs of adhesive and HDPE (indicated as fluids A and B, respectively) for operating points 1–6 (listed in Table 7) and calculated the dimensionless position of the interface κ using the model described in Section 3.3. For this purpose, the dimensionless input quantities for the model were derived for each operating point by:

- Evaluation of the Tait equation (Equation (3)) for each melt on the basis of melt temperature and average pressure within the co-extrusion flow region;
- Transformation of individual and total mass throughputs into corresponding volumetric flow rates;
- Evaluation of average flow velocity and representative shear rate according to Equations (7) and (9);
- Calculation of local power-law parameters from Carreau-Yasuda model parameters for each melt using Equations (10) and (11);
- Calculation of dimensionless input quantities χ and $\Pi_{V}^{A}$ using Equations (6) and (8);
- Evaluation of the κ-model (Equation (5)).

**Figure 8:**
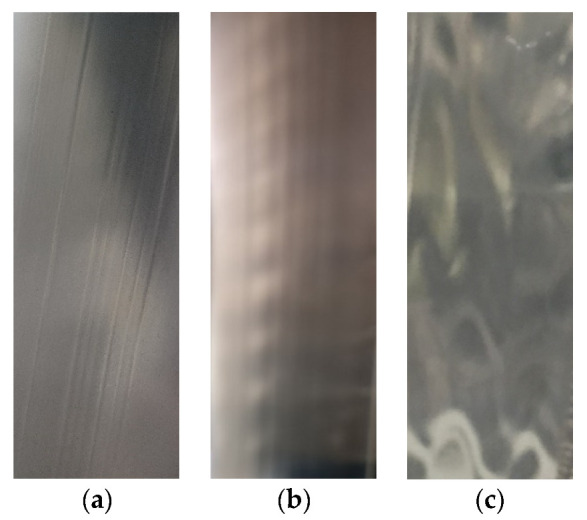
Images of the adhesive-HDPE co-extrudate in the molten state directly after exiting the die for various operating points showing (**a**) stable flow behavior, (**b**) unstable flow behavior (wave-type instabilities), and (**c**) highly unstable flow behavior (zig-zag-type instabilities). The image areas displayed comprise a size of approximately 80 mm × 30 mm each.

The final results for the dimensionless position of the interface are given in Table 7. Furthermore, we assigned the class labels stable (+), transition regime (~), unstable (-), and highly unstable (--) to the operating points by visual assessment of the co-extrudate quality. Note that unstable flow behavior correlates to wave-type instabilities and, for highly unstable flows, interfacial distortions of the zig-zag type are observed. Figure 8 shows the adhesive-HDPE co-extrudate for three different operating points characterized by (a) stable flow behavior, and interfacial instabilities of (b) the wave type (unstable) and (c) the zig-zag type (highly unstable). Visually assessing all operating points under investigation gave the following results: operating points 1 and 2 were considered to be stable, wave formation commenced at the interface for operating points 3 and 4 (transition), unstable behavior (waves) was observed at operating point 5, and severe distortions (zig-zag defects) as shown in Figure 8c were detected for operating point 6. Hence, it can be concluded that for a total throughput of approximately 2.80 kg·h^−1^, shifting the interface from the center towards the upper die wall changed the flow situation from stable to incipient unstable flow behavior. Increasing the total throughput and dimensionless interface position even intensified process instabilities (wave and zig-zag type). Nevertheless, for unbiased and reproducible assessment of interlayer flow instabilities and their quantification, an in situ detection system with proper data evaluation is indispensable. Visual observation of the co-extrudate is also limited to transparent melts.

### 4.1. OCT Measurements

To precisely determine the thickness of an object by OCT knowledge of the material’s refractive index to convert the optical depth between two interfaces into real depth values is required. In our particular application, the refractive indices of the melts were identified based on a monolayer polymer melt flow through the die that was realized by feeding both extruders with the same material. A total of 600 A-scans forming the B-scan were summed over the scan width, and the two main peaks that indicate the glass-melt and melt–steel interfaces were identified. The refractive index n of the polymer melt was then obtained by computing the ratio between the optical distances of the two peaks $l_{opt}$ and the defined height of the flow channel $l_{real}$:

(20) $$n=\frac{l_{opt}}{l_{real}}.$$

Table 8 shows the results for the refractive indices of the adhesive and the HDPE at a wavelength of 1300 nm in terms of mean and standard deviation for five measurements per material. Note that the refractive indices of the materials investigated depend on the phase state (amorphous in the molten state and semicrystalline in the solid state) and the wavelength of the incident light beam and, thus, the values obtained are slightly below those commonly reported for polymers (e.g., 1.52 for HDPE [45]). Since the adhesive is based on polyethylene, its refractive index differs only minimally from that of HDPE.

**Figure 9:**
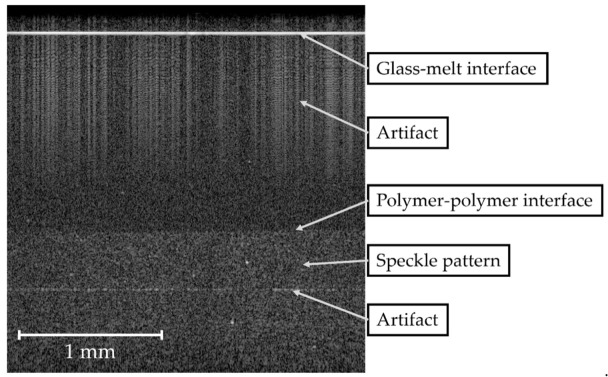
Image of an OCT measurement (B-Scan) during co-extrusion, showing glass-melt and polymer-polymer interfaces.

To determine the interface position as a function of time, B-scans of the OCT measurements during co-extrusion, as presented in Figure 9, were recorded. With an optical measurement range of *l_range_* = 2.54 mm and a resolution of *N_pixel_* = 512 pixel in the vertical direction, the melt-glass and polymer-polymer interfaces were detected. Noise and artifacts that interfere with the polymer-polymer interface signals were to be prevented while recording measurement data.

For further analysis, MATLAB [46] was used to register the polymer-polymer interface position by evaluating the set of consecutive B-scans. To this end, the scans were transformed into greyscale images. After defining a region of interest in the image, the interface was selected manually in the first scan and, using a MATLAB algorithm, was determined automatically in the subsequent B-scans. The interface was thus given by the pixel position $N_{pos,pixel}$ outgoing from the upper edge of the B-scan image in the vertical direction. To convert a pixel position into a real length value $l_{real}$ for the fluctuation of the interface, the optical length value $l_{opt}$ was calculated first by:

(21) $$l_{opt}=N_{pos,pixel}\frac{l_{range}}{N_{pixel}}.$$

The real depth value was then obtained by the quotient of the optical depth value and the refractive index n:

(22) $$l_{real}=\frac{l_{opt}}{n}.$$

The real depth position of the interface was then determined as a function of time, and the following evaluation parameters were tested to assess their significance in detecting and quantifying co-extrusion instabilities:

- Standard deviation;
- Signal intensity;
- Number of zero-crossings;
- Amplitude of main frequency by fast Fourier transform (FFT).

**Figure 10:**
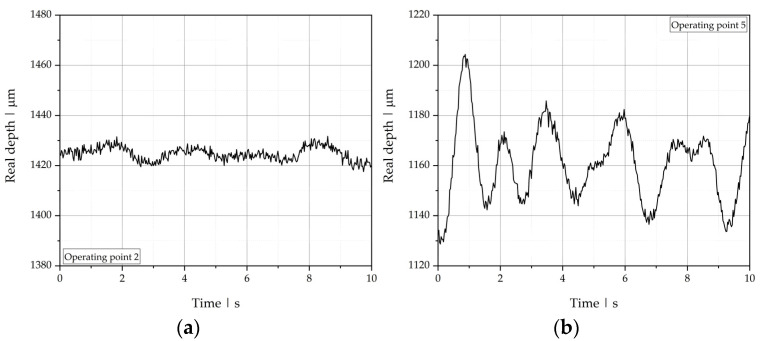
Real depth position of the polymer-polymer interface over time for (**a**) operating point 2 and (**b**) operating point 5.

To illustrate the evaluation procedure, we use the OCT measurement results at two operating points that were visually classified as stable (operating point 2) and unstable with wave-type instabilities (operating point 5) as examples. In the first step of OCT data analysis, we plotted the real depth position (discretized into k measurements) over time (Figure 10) and calculated its mean value $\bar{l_{real}}$ and standard deviation $\sigma_{l_{real}}\;$ according to:

(23) $$\bar{l_{real}}=\frac{1}{k}\overset{k}{\underset{i=1}{\sum }}l_{real,i}$$

(24) $$\sigma_{l_{real}}=\sqrt{\frac{\sum_{i=1}^{k}\left(l_{real,i}-\bar{l_{real}}\right)}{k}}.$$

The signals were then shifted by the mean:

(25) $$l_{shifted}=l_{real}-\bar{l_{real}}.$$

**Figure 11:**
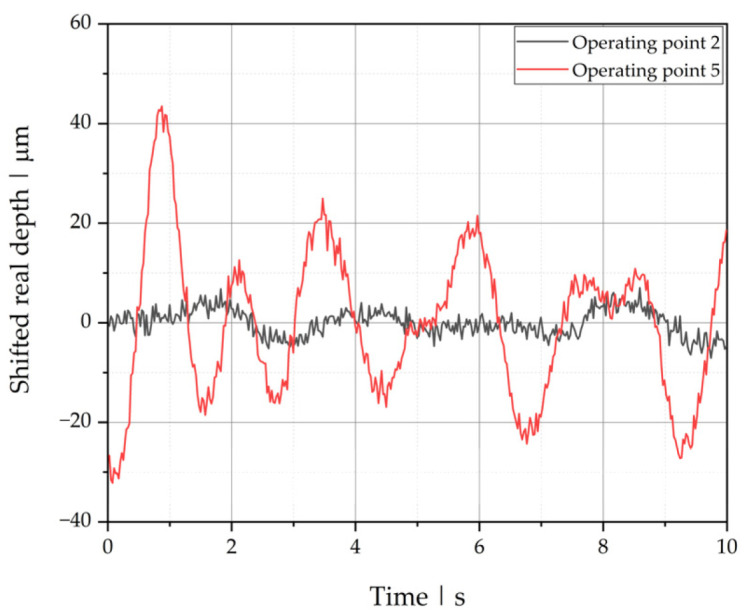
Real depth position of the polymer-polymer interface over time, shifted by the mean, for operating points 2 (stable) and 5 (unstable).

Figure 11 shows the fluctuation of the interface for the unstable operating point 5 compared to the stable operating point 2. Stable and unstable process points can be distinguished directly by means of their standard deviations. A comparison of all operating points using the standard deviation as classification is presented at the end of this section.

**Figure 12:**
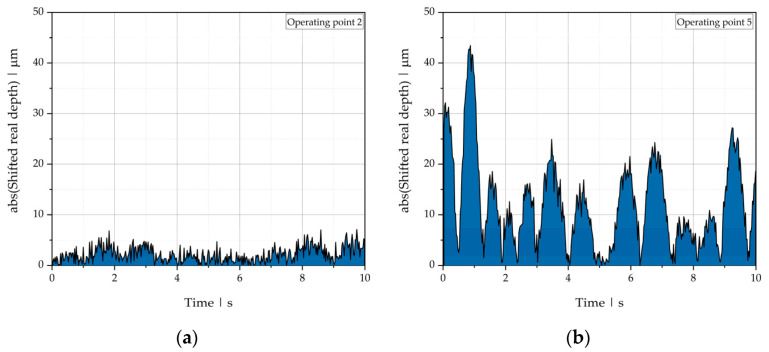
Signal intensities for absolute values of real length position of the polymer-polymer interface over time, shifted by the mean, for (**a**) operating point 2 and (**b**) operating point 5.

To determine the signal intensity, the absolute values of the shifted data points were taken, and the resulting curve was integrated, as illustrated in Figure 12. For integration, a trapezoidal rule was used:

(26) $$\overset{t_{end}}{\underset{0}{\int }}l_{real}\left(t\right)dt\;\approx \;\overset{N}{\underset{i=1}{\sum }}\frac{l_{real}\left(t_{k}-1\right)-l_{real}\left(t_{k}\right)}{2}\Delta t_{k}.$$

**Figure 13:**
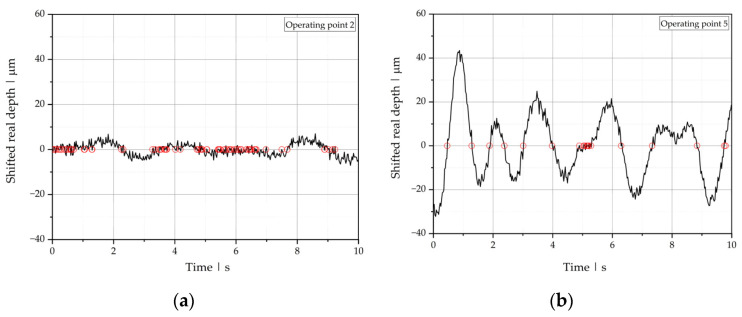
Number of zero-crossings of the real depth position of the polymer-polymer interface over time, shifted by the mean, for (**a**) operating point 2 and (**b**) operating point 5.

The number of times the interface signal of each operating point crossed zero was counted (Figure 13). It can be seen that, with instabilities, the signal intensity is higher, and the number of zero-crossings lower.

**Figure 14:**
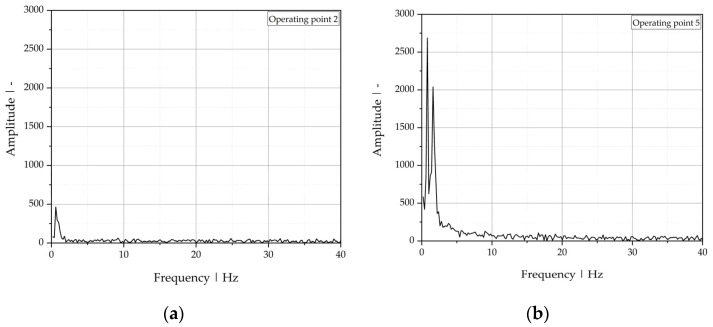
Fast Fourier transform of the real length position of the polymer-polymer interface over time, shifted by the mean, for (**a**) operating point 2 and (**b**) operating point 5.

In a further evaluation step, the depth position signal was transferred from the time domain to the frequency domain using fast Fourier transform, as shown in Figure 14. Comparing the magnitudes of the main frequencies of the depth signals allows distinguishing between operating points 2 and 5 (stable and unstable behavior, respectively).

**Figure 15:**
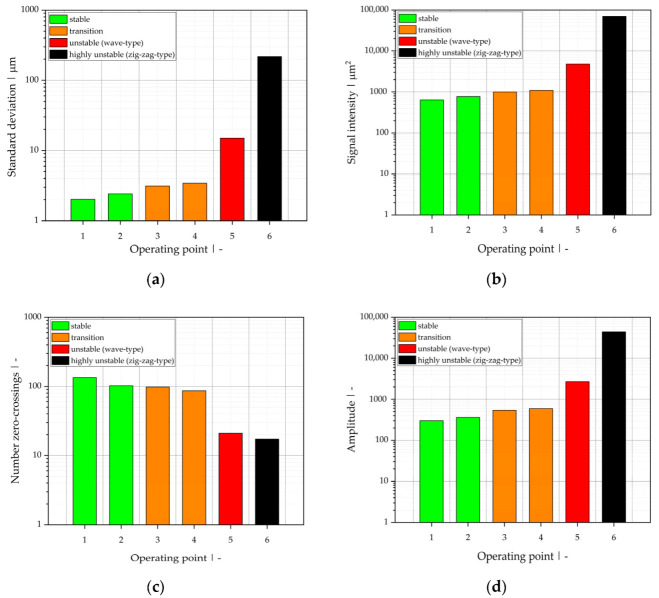
Quantitative values of (**a**) standard deviation, (**b**) signal intensity of measurement data, (**c**) number of zero-crossings, and (**d**) magnitude of the main frequency resulting from FFT for operating points 1–6. Process stability is indicated by the color of the bars: stable (green), transition (orange), unstable (red), and highly unstable (black).

To compare the results for all operating points listed in Table 7, quantitative values of the defined evaluation parameters are plotted in Figure 15. In accordance with the visual evaluation, the operating points are classified as stable, transition, unstable (wave-type instability), and highly unstable (zig-zag-type instability). The results of OCT measurements and visual evaluation are in good agreement. Unlike for the stable operating points 1 and 2, small oscillations of the interface (beginning formation of waves) can be observed for operating points 3 and 4, which are clearly separable, but result in slightly higher standard deviations, signal intensities, and amplitudes of the main frequency, as well as slightly smaller numbers of zero-crossings. Thus, OCT not only offers the possibility of directly detecting the interfacial position between melts with similar refractive indices, but also enables the detection of even marginal oscillations of it, which can be explained by the depth resolution of the sensor used and the choice of evaluation parameters.

**Figure 16:**
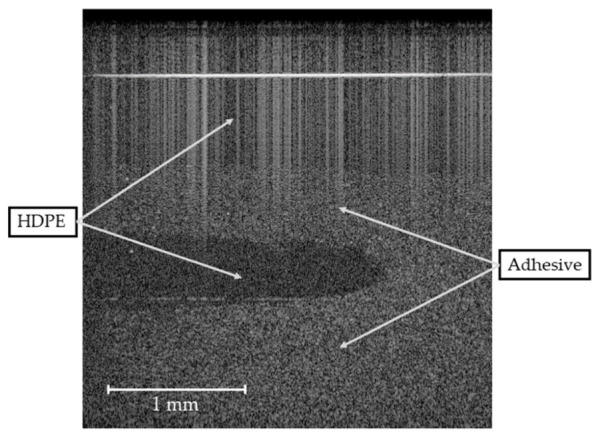
B-scan showing interpenetration of adhesive and HDPE, which leads to mixing of the polymers at the interface and to local formation of multiple layers at the highly unstable operating point 6.

The same results were obtained for the unstable (wave-type instability) and highly unstable (zig-zag instability) operating points, but the distinction based on the parameters evaluated is even more pronounced. Operating point 6, classified as highly unstable, is also characterized by interpenetration of the polymer melts, which leads to a local mixing effect at the interface (see B-scan in Figure 16).

Based on the results of OCT measurements in combination with visual co-extrudate assessment, we conclude that all evaluation parameters mentioned are suitable to classify operating points into stable, transition, unstable, and highly unstable. We propose using upper and lower limits as listed in Table 9 for these class labels.

### 4.2. Ultrasonic Measurements

**Figure 17:**
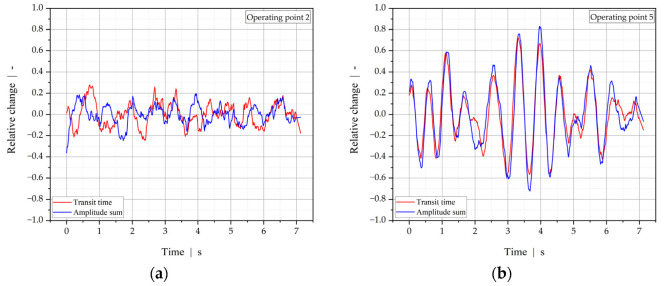
Relative change in transit time and amplitude sum (intensity) at (**a**) operating point 2 and (**b**) operating point 5 in the ultrasound detection of flow instabilities when co-extruding HDPE and an adhesive layer.

Among the ultrasound techniques, the method of correlating the relative change in ultrasound transit time with the relative change in signal intensity yielded the best results for the unbiased assessment and quantification of interfacial distortion in the co-extrusion of melts of similar acoustic impedances. Figure 17 plots the relative change in transit time and the amplitude sum (intensity) versus measurement time for the example operating points 2 and 5. Operating point 5 is characterized by a significantly greater variation in the relative changes in transit time and intensity, which may be due to greater variations in the interface position and, thus, more significant variations in the ratio of impedances. Additionally, the linear coefficient of correlation between the two signals was found to be 0.39 for operating point 2 and 0.94 for operating point 5, which indicates that for operating point 2 signal interaction was low and, thus, stable flow behavior can be assumed, while for operating point 5 the correlation was strong and the process unstable. These results are in good agreement with intuitive visual assessment of the co-extrudate and results of the OCT measurement.

**Figure 18:**
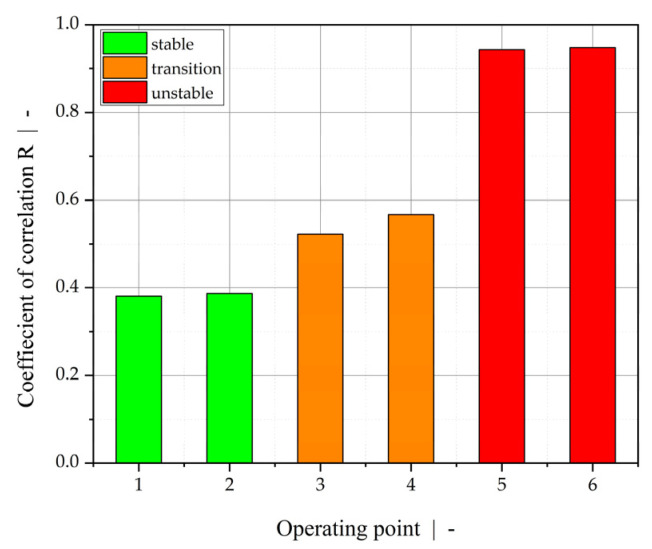
Coefficient of correlation between transient time and amplitude sum for operating points 1–6, based on ultrasound measurement. Process stability is indicated by the color of the bars: stable (green), transition (orange), and unstable/highly unstable (red).

Furthermore, we evaluated the linear coefficients of correlation for all operating points and assigned stable and unstable flow behavior. In the literature [47], coefficients of correlation are commonly grouped into (1) low interaction, (2) average interaction, and (3) strong interaction for values smaller than 0.3, between 0.3 and 0.5, and greater than 0.5, respectively. Based on this scale, we assessed operating points 1–6 in terms of interfacial instabilities, as summarized in Figure 18. Operating points 1 and 2 are considered to be stable (green bar), operating points 3 and 4 are in the transition regime (orange bar), and operating points 5 and 6 are unstable (red bar). The results obtained are in good accordance with visual observations and OCT measurements. However, closer investigation of unstable process behavior by differentiating between wave-type instabilities and zig-zag-type interlayer distortion is not possible using the ultrasound approach.

### 4.3. Comparison of the Measurement Approaches

Both OCT and ultrasound techniques allow assessment of the interfacial stability in the co-extrusion flow of an adhesive layer and a HDPE melt. Choosing suitable parameters in the signal evaluation enables the level of instability of the flow to be quantified using the measurement approaches presented. In OCT measurement, the standard deviation, intensity, number of zero-crossings, and main frequency of the time-dependent oscillation of the interface could be correlated with the stability of operating points (according to visual observations). Comparable results were obtained by correlating the transit time and amplitude of the signal of the ultrasonic measurement in transmission mode. In our particular measurement task, OCT and ultrasound technology have distinct advantages and disadvantages, which are summarized and compared to visual assessment in Table 10. By producing contactless B-scans, the OCT method offers a means of generating two-dimensional cross-sectional images of the co-extrusion flow as it evolves over time; it therefore allows direct observation and recording of the interface between the polymers and its absolute position within the flow channel. OCT further enables interface detection for a wide range of polymer-polymer combinations, since the majority show high transmittance at the relevant wavelengths. Additionally, even extremely small differences in refractive indices are sufficient to distinguish between different materials (see the example combination of adhesive and HDPE). All operating points in this work were reproducible in terms of pressure and temperature data by either using the massive steel cover or the cover with the glass insert, which proves that the effect of the glass insert for OCT measurements on the co-extrusion flow can be omitted for the material combination investigated. The ultrasound technique, in contrast, is much less expensive and requires no optical viewport (which would also constrain the measurement position), but does require direct clamping of the probe to the steel die (which may affect signal quality and, thus, reproducibility). In summary, both methods for the in situ detection of co-extrusion interfacial flow instabilities outperform simple visual extrudate assessment, since they enable an unbiased characterization of the interface and quantification of its instability.

## 5. Conclusions

In this study, we developed two methods for the in situ detection of interfacial instabilities in two-layer co-extrusion flows by applying optical coherence tomography (OCT) and ultrasound (US) sensor technology. Both approaches are independent of ambient conditions and the subjectivity of human observers. Furthermore, we defined suitable parameters for signal evaluation to quantify the level of interface distortion. For OCT, the standard deviation of the interface position as a function of time, the signal intensity with respect to the average interface position, the number of zero-crossings with respect to the average interface position, and the magnitude of the main frequency of the FFT signal were identified as being suitable. For US, the correlation coefficient between the relative changes in amplitude and transit time in the transmission mode provides a practical measure. On this basis, we investigated operating points with various ratios of individual throughputs and overall mass throughputs, which were categorized into different classes, including stable process behavior, a transition regime, and unstable performance (characterized by either wave or zig-zag instabilities). As expected, the co-extrusion flow transforms from stable to unstable by shifting the interfacial position towards a die wall (assuming a given overall throughput) and/or the flow rate increases further. This result is also in good accordance with observations in the literature (e.g., Schrenk et al. [5] proposing a critical interfacial shear stress in co-extrusion). However, this work focuses on the development of an in situ detection and quantification method for flow instabilities; thus, the measurement concepts based on OCT and US with their evaluation procedures were successfully tested to classify the operating points into the above-mentioned categories. Generation of B-scans by OCT enables additional distinction between wavy instabilities and local convective mixing (zig-zag instabilities) at the interface for unstable flows. Since the reasons for interfacial flow instabilities in co-extrusion processes are not completely resolved, we are planning on comprehensive and systematic investigations on the basis of the developments in this work.

In summary, the choice of sensor technology highly depends on the aims of the investigation, since the two approaches differ in (1) the determination of flow instabilities (direct or indirect), (2) the possibility of determining the absolute interface position, (3) the flexibility of the measurement position, (4) the engineering requirements imposed on the co-extrusion die, and (5) the investment costs. However, in contrast to visual observation, both technologies provide objective measures for characterizing the flow condition, and enable real-time in situ process monitoring. Subsequently, they will contribute to die design, process settings, and layer arrangement in order to avoid interfacial defects.

Developed for a two-layer co-extrusion flow, OCT and US are also directly applicable to multilayer flows. OCT requires a difference in the refractive index or in the speckle pattern, and US requires differences in the attenuation coefficient α and in the longitudinal sound velocity $c_{L}$ at the interface investigated. Since, in this work, two very similar polymers were used, this condition can be assumed to be fulfilled for almost all interfaces. For OCT, the interface under investigation must be accessible from at least one side of the multilayer structure for the wavelength used.

## Figures and Tables

**Table 1:** Supplier, main application, and properties of the materials investigated.

Type	Grade	Supplier	Application	Density (ISO 1183)	MFR (ISO 1133)
HDPE	Hostalen ACP5831D	Lyondell Basell	Blow molding	0.958 g·cm^−3^	0.25 g/10 min (190 °C/2.16 kg)
Adhesive	Admer NF408E	Mitsui Chemicals	Adhesive resin	0.920 g·cm^−3^	1.60 g/10 min (190 °C/2.16 kg)

**Table 2:** Carreau-Yasuda parameters for HDPE ACP5831D and adhesive NF408E.

Parameter	Unit	HDPE ACP5831D	Adhesive NF408E
$n_{CY}$	-	1.057 × 10^−4^	1.572 × 10^−4^
*a*	-	3.982 × 10^−1^	4.876 × 10^−1^
$\eta_{0}$	Pa·s	3.474 × 10^4^	6.060 × 10^3^
$\eta_{\infty }$	Pa·s	0	0
λ	s	1.203 × 10^−1^	1.029 × 10^−2^
α	-	1.224 × 10^−2^	1.633 × 10^−2^
*T*_0_	K	473.15	473.15

**Table 3:** Tait parameters for HDPE ACP5831D and adhesive NF408E.

Parameter	Unit	HDPE ACP5831D	Adhesive NF408E
$b_{1m}$	m^3^·kg^−1^	1.43 × 10^−3^	1.39 × 10^−3^
$b_{2m}$	m·(kg·K)^−1^	6.18 × 10^−7^	6.18 × 10^−7^
$b_{3m}$	Pa	6.00 × 10^7^	6.00 × 10^7^
$b_{4m}$	K^−1^	6.49 × 10^−5^	6.49 × 10^−5^
$b_{5m}$	K	622.28	573.15

**Table 4:** Barrel, adapter, and die temperatures.

Material	Unit	B1	B2	B3	A	T_Die_
HDPE ACP581D	°C	180	190	190	190	200
Adhesive NF408E	°C	190	200	200	200	

**Table 5:** Overview of the operating points under investigation (screw rotational speeds).

Operating Point	Screw Speed | rpm	
	Adhesive NF408E	HDPE ACP5831D
1	14.2	12
2	16.2	9.8
3	17.0	9.0
4	17.5	8.5
5	28.0	8.0
6	48.0	8.0

**Table 6:** Specifications of the spectral domain OCT system.

Parameter	Unit	Value
Center wavelength	nm	1300
Line rate (A-scan rate, typical)	kHz	28
Axial resolution (depth resolution)	µm	7.5
Lateral resolution	µm	15
Maximum field of view	mm	10 × 10 × 2.54
Maximum pixels per A-scan	-	512
Sensitivity (typical)	dB	102

**Table 7:** Overview of mass throughputs, dimensionless position of the interface, and visual assessment of flow instabilities of the co-extrudate for the operating points under investigation.

Operating Point	Mass Throughput | kg·h^−1^			Position of Interface κ | -	Stability | +/~/-/--
	Adhesive NF408E	HDPE ACP5831D	Total	Adhesive HDPE	Adhesive HDPE
1	1.62	1.18	2.80	0.523	+
2	1.84	0.97	2.80	0.571	+
3	1.94	0.88	2.81	0.593	~
4	1.99	0.82	2.82	0.606	~
5	3.16	0.74	3.90	0.691	-
6	5.35	0.71	6.06	0.763	--

**Table 8:** Refractive indices of the adhesive and the HDPE melt at a wavelength of 1300 nm.

Material	Refractive index | -	
	Mean	STD
Adhesive NF408E	1.375	0.00109
HDPE ACP581D	1.387	0.00194

**Table 9:** Upper and lower limits of the parameters evaluated for stability classification.

Class Label	Standard Deviation | µm		Signal Intensity | µm^2^		Number of Zero-Crossings | -		Magnitude Main Frequency | -	
	Min.	Max.	Min.	Max.	Min.	Max.	Min.	Max.
Stable		<3		<1000	>100			<500
Transition	3	6	1000	2500	30	100	500	1000
Unstable	6	50	2500	20,000	15	30	1000	10,000
Highly unstable	>50		>20,000			<15	>10,000	

**Table 10:** Summary of the advantages and features of the detection methods investigated.

Parameter	OCT	Ultrasonic	Visual
Determination of interface position	Possible; requires refractive indices of melts	Not possible	Not possible
Measurement position	Optical viewport required	Flexible	Assessment of co-extrudate at die outlet
Coupling of sensor probe	Contactless	Direct coupling to steel body required	-
Limitations in terms of material properties	Optical transparency at wavelength of OCT required; high sensitivity regarding differences in optical properties	Indirect measurement necessary because impedances of polymers are often highly similar	Transparent materials required (at wavelength of visible light)
Investment costs	High	Low	None
Potential for integration into industrial processes	Robust, unbiased results, real-time evaluation possible	Robust, unbiased results, real-time evaluation possible	Observer-dependent results
Data amount	High (B-scans, depends on sampling rate)	Medium (depends on sampling rate)	None; no direct quantification possible

## Data Availability

The data presented in this study are available on request from the corresponding author.
